# Low Levels of MicroRNA-10a in Cardiovascular Endothelium and Blood Serum Are Related to Human Atherosclerotic Disease

**DOI:** 10.1155/2021/1452917

**Published:** 2021-07-15

**Authors:** Jong-Tar Kuo, Hsiao-En Tsai, Ching-Ting Lin, Chih-I Lee, Pei-Ling Lee, Yu-Rong Ruan, Jeng-Jiann Chiu, Ding-Yu Lee

**Affiliations:** ^1^Department of Biological Science and Technology, China University of Science and Technology, Taipei, Taiwan; ^2^Division of Cardiovascular Surgery, Department of Surgery, National Taiwan University Hsin-Chu Hospital, Hsinchu, Taiwan; ^3^Graduate Institute of Clinical Medicine, National Taiwan University College of Medicine, Taipei, Taiwan; ^4^School of Chinese Medicine, China Medical University, Taichung, Taiwan; ^5^Institute of Cellular and System Medicine, National Health Research Institutes, Miaoli, Taiwan

## Abstract

**Background:**

MicroRNA-10a (miR-10a) inhibits transcriptional factor GATA6 to repress inflammatory GATA6/VCAM-1 signaling, which is regulated by blood flow to affect endothelial function/dysfunction. This study aimed to identify the expression patterns of miR-10a/GATA6/VCAM-1 *in vivo* and study their implications in the pathophysiology of human coronary artery disease (CAD), i.e., atherosclerosis.

**Methods:**

Human atherosclerotic coronary arteries and nondiseased arteries were used to detect the expressions of miR-10a/GATA6/VCAM-1 in pathogenic *vs*. normal conditions. In addition, sera from CAD patients and healthy subjects were collected to detect the level of circulating miR-10a.

**Results:**

The comparison of human atherosclerotic coronary arteries with nondiseased arteries demonstrated that lower levels of endothelial miR-10a are related to human atherogenesis. Moreover, GATA6/VCAM-1 (a downstream target of miR-10a) was highly expressed in the endothelium, accompanied by the reduced levels of miR-10a during the development of human atherosclerosis. In addition, CAD patients had a significantly lower concentration of miR-10a in their serum compared to healthy subjects.

**Conclusions:**

Our findings suggest that low miR-10a and high GATA6/VCAM-1 in the cardiovascular endothelium correlates to the development of human atherosclerotic lesions, suggesting that miR-10a signaling has the potential to be developed as a biomarker for human atherosclerosis.

## 1. Introduction

Vascular endothelial cells (ECs) modulated by shear stress resulting from blood flow play vital roles in cardiovascular pathology [1]. The blood shear stresses, i.e., oscillatory shear (OS) and pulsatile shear stress (PS), activate different mechanotransduction pathways to modulate EC dysfunction and function [1]. OS occurs in bifurcation and curvature sites (the atherosclerosis-susceptible regions) of the cardiovascular system and serves as “pathogenic shear” to induce EC inflammatory signaling and promote the development of atherosclerosis. In contrast, PS occurs in straight regions (the atherosclerosis-resistant regions) of the cardiovascular system and serves as a “protective shear” to inhibit EC inflammatory signaling and protect blood vessels from atherosclerosis [1–4].

MicroRNAs (miRs) are the noncoding RNA molecules that bind to the 3′-untranslated regions (3′-UTR) of the target mRNAs and mediate RNA silencing and regulate gene expression. Reportedly, miRs regulate several biological processes, including regulating cardiovascular functions [5–7]. Moreover, studies have shown that hemodynamic shear stress regulates the expressions of miRs promoting EC function or dysfunction; in particular, OS-modulated miRs promote the development of atherosclerosis [5, 8–10]. An miR microarray study in an *in vivo* experimental swine model demonstrated that EC miR-10a is a vital shear-regulated miR with the lowest relative expression among all miRs in the pathogenic OS region (atherosclerosis-susceptible regions) of the cardiovascular system [11]. Moreover, the potential of miR-10a to inhibit inflammatory signaling in ECs is also evident from *in vitro* and *in vivo* studies [11, 12]. *In vitro* studies have demonstrated that the activation of NF-*κ*B and the expression of inflammatory molecules are induced by the inhibition of EC miR-10a [11]. Furthermore, in a previous study, we have demonstrated that miR-10a directly binds to the 3′-UTR of GATA6 (an inflammatory transcriptional factor) to abolish its expression, consequently inhibiting the downstream vascular cell adhesion molecule (VCAM-1) [12]. The study has also reported that PS induces miR-10a expression to repress inflammatory GATA6/VCAM-1 signaling in ECs, whereas OS inhibits miR-10a expression to enhance inflammatory GATA6/VCAM-1 signaling [12]. Taken together, these studies suggest that the hemodynamic shear stresses, i.e., PS and OS, have differential effects on the alteration of miR-10a expression to turn on the anti-inflammatory and inflammatory signaling pathways, respectively [12]. Recently, we used an ApoE knockout (ApoE-/-) mouse model to show that endothelial miR-10a can be induced by precursor miR-10a or RAR*α*/RXR*α* agonists to inhibit GATA6/VCAM-1 signaling, inflammatory cell infiltration, and atherosclerotic lesion formation [12–14]. Despite several *in vitro* and *in vivo* reports on the anti-inflammatory effect of shear-sensitive miR-10a in ECs, the role of endothelial miR-10a/GATA6/VCAM-1 signaling during the progression of human atherosclerosis remains unclear *in vivo*. In addition, the clinical applications of miR-10a in human atherosclerotic disease still need to be identified.

This present study aims to identify the expression patterns of miR-10a/GATA6/VCAM-1 *in vivo* and study their implications in the pathophysiology of human coronary artery disease (CAD). Here, we compared the diseased human coronary arteries with nondiseased human arteries, i.e., human coronary arteries with advanced atherosclerotic lesion vs. human coronary arteries with minimal atherosclerotic lesion or nondiseased human internal thoracic aorta, to identify the *in vivo* role of miR-10a/GATA6/VCAM-1 signaling in pathogenic vs. normal condition in the human cardiovascular system. Furthermore, we correlated expression patterns of miR-10a and GATA6/VCAM-1 in the endothelium with the development of human atherosclerotic lesions. In addition, we investigated the relative levels of miR-10a in the sera collected from human blood from patients with CAD and healthy subjects.

## 2. Materials and Methods

### 2.1. Diseased Human Coronary Arteries and Nondiseased Human Arteries

Eight diseased human coronary arteries were harvested from five end-stage heart failure patients undergoing heart transplantation at Tri-Service General Hospital in Taiwan. The study was carried out in accordance with the Declaration of Helsinki and approved by the Institutional Review Board of the Tri-Service General Hospital. Informed consent was obtained from all participants. These arteries contained various stages of atherosclerotic lesions, from minimal lesions (mini. lesion, with no intimal thickening and intimal macrophages) to advanced lesions (advan. lesion, with increased intima thickness and intimal macrophages). Nondiseased internal thoracic arteries obtained from four CAD patients undergoing coronary artery bypass graft surgery (CL artery) were used as control arteries.

### 2.2. Detection of miR-10a, GATA6, and VCAM-1 Expressions

Coronary arteries with advan. lesions and mini. lesions and CL arteries were fixed in formalin and embedded in paraffin blocks. The expression of miR-10a in the cross-sections of these samples was estimated by miR in situ hybridization. Furthermore, we estimated the expression levels of GATA6 and VCAM-1 by immunohistochemical staining.

#### 2.2.1. MiR In Situ Hybridization to Detect miR-10a Expression

The miR-10a expression in the samples was determined by fluorescence in situ hybridization [15], as described previously [13]. In brief, cross-sections of coronary arteries with advan. lesions and coronary arteries with mini. lesions or nondiseased arteries were incubated with 5′DIG-labeled locked-nucleic acid probes (20 nmol/L) (Thermo Scientific, MA) at an optimal temperature. The cross-sections were further incubated with HRP-conjugated antibody against DIG (Roche Applied Science, Germany), and the signal was amplified using TMR-tyramide (Perkin Elmer, MA). The slides were mounted, and the images were captured using a fluorescence microscope.

#### 2.2.2. Immunohistochemical Staining to Detect the Expressions of GATA6 and VCAM-1

Immunohistochemical staining was performed as previously described [13]. Two serial sections were subjected to immunohistochemical staining for detecting GATA6 or VCAM-1 and the EC marker (i.e., von Willebrand factor (vWF)), respectively. In brief, serial sections (4 *μ*m thick) of coronary and internal thoracic arteries were deparaffinized and then blocked for 1 h with serum albumin dissolved in phosphate-buffered saline (5 mg/mL). One section was incubated with anti-GATA6 antibody (Santa Cruz, CA) or anti-VCAM-1 antibody (Santa Cruz, CA) (1 : 50) for 1 h at 37°C, followed by rhodamine-conjugated secondary antibody (1 : 1000) in the presence of DAPI for 2 h at room temperature. The second section was incubated with an anti-vWF antibody (Santa Cruz, CA) (1 : 50), followed by the secondary antibody for 2 h at room temperature. The slides were mounted, and the images were captured using a fluorescence microscope.

### 2.3. Blood Serum Collection, RNA Extraction, and miR-10a Detection

The serum level of miR-10a was assessed following the procedures described in a previous study [13]. Fresh blood from human subjects without (*n* = 13) or with (*n* = 30) CAD was collected into 10 mL BD Vacutainer K3EDTA tubes (BD Biosciences, MA). The study and experimental design was approved by the Ethics Review Board of the National Health Research Institutes, and informed consent was obtained from all participants. Blood serum was collected by centrifugation at 2500x g for 10 min at room temperature and then stored at −80°C. Total RNA was further isolated using TRIzol reagent (Thermo Scientific) and supplemented with 5 nmol/L *Caenorhabditis elegans* miR-39 (cel-miR-39) (Thermo Scientific) as Spike-in Control, as per the protocol described previously [16]. The levels of miR-10a in these sera were measured using RT-qPCR with iQ SYBR Green supermix (Bio-Rad).

## 3. Results

### 3.1. Low miR-10a Level in the Endothelium Is Related to the Progression of Atherosclerotic Lesions in Human Coronary Arteries In Vivo

**Figure 1:**
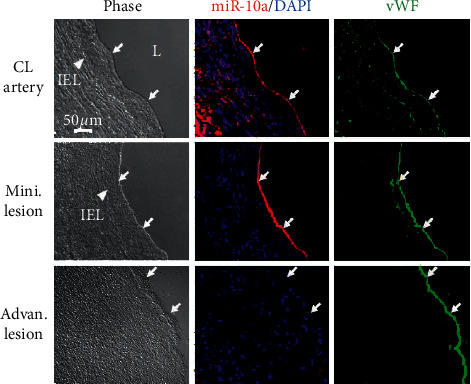
miR-10a is highly expressed in the EC layer of human nondiseased arteries but is decreased in atherosclerotic coronary arteries. Cross-section samples of coronary arteries with advanced atherosclerotic lesion (advan. lesion) vs. coronary arteries with minimal atherosclerotic lesion (mini. lesion) or nondiseased internal thoracic aorta (CL artery) were stained with human miR-10a and vWF (i.e., EC marker) and counterstained with DAPI. vWF was used to identify the endothelium of blood vessels. Arrow, vWF-positive ECs; IEL, internal elastic lamina; *L*, lumen; EC, endothelial cell; CL artery, control artery.

The results of miR in situ hybridization show pronounced staining of miR-10a in the endothelium of nondiseased CL artery (Figure 1, top row) and coronary arteries with mini. lesion (Figure 1, middle row). However, the expression of miR-10a was rare in the endothelium of diseased coronary arteries with advan. lesion (Figure 1, bottom row). These results suggest that low miR-10a levels in the endothelium could be related to the development of atherosclerotic lesions in human coronary arteries in vivo.

### 3.2. GATA6 (Direct Target of miR-10a) Is Highly Expressed in the Endothelium of Human Atherosclerotic Coronary Arteries In Vivo

**Figure 2:**
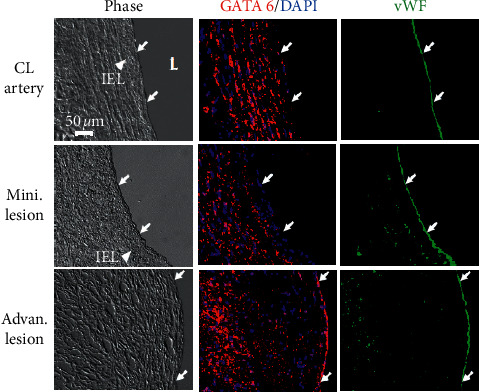
GATA6 (direct target of miR-10a) in the endothelium is highly expressed in human atherosclerotic coronary arteries *in vivo*. Cross-sections of diseased human arteries vs. nondiseased human arteries, i.e., coronary arteries with advan. lesions vs. coronary arteries with mini. lesions or CL arteries, were immunohistochemically stained to detect inflammatory transcriptional factor GATA6 and vWF and counterstained with DAPI. vWF was used to identify the endothelium of blood vessels. Arrow, vWF-positive endothelial cells; IEL, internal elastic lamina; L lumen; CL artery, control artery.

Our previous in vitro study demonstrated that the transcriptional factor GATA6 is the direct target of miR-10a, and that its expression can be inhibited by miR-10a through its binding to the 3′-UTR of GATA6 [12]. Here, we used immunohistochemical staining to investigate the in vivo expression patterns of GATA6 in the endothelia of nondiseased human arteries and diseased human coronary arteries. As shown in Figure 2, GATA6 is highly expressed in the endothelium of diseased coronary arteries with advan. lesions (Figure 2, bottom row). In contrast, the expression of GATA6 was not detected in the endothelium of the CL artery (Figure 2, top row) and coronary arteries with mini. lesions (Figure 2, middle row). These results suggest that GATA6, the direct target of miR-10a is highly expressed in the endothelium of human atherosclerotic coronary arteries in vivo.

### 3.3. VCAM-1 (a Downstream Target of miR-10a) Is Upregulated in the Endothelium of Human Atherosclerotic Coronary Arteries In Vivo

**Figure 3:**
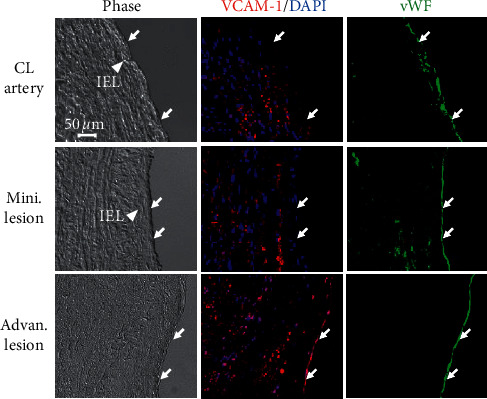
miR-10a downstream VCAM-1 in the endothelium is upregulated in human atherosclerotic coronary arteries *in vivo*. Cross-sections of diseased coronary arteries with advan. lesions vs. coronary arteries with mini. lesions or CL arteries were immunohistochemically stained for the inflammatory molecule VCAM-1 and vWF and counterstained with DAPI. vWF was used to identify the endothelium of blood vessels. Arrow, vWF-positive endothelial cells; IEL, internal elastic lamina; L lumen; CL artery, control artery.

The inflammatory molecule, VCAM-1, regulated transcriptionally by the transcriptional factor GATA6, has been identified as a downstream molecule of miR-10a and GATA6 [12, 17]. In this study, we showed that the expression of VCAM-1 was upregulated in the endothelium of diseased coronary arteries with advan. lesions (Figure 3, bottom row), whereas it was downregulated in the endothelium of the CL arteries (Figure 3, top row) and coronary arteries with mini. lesions (Figure 3, middle row). Furthermore, the expression patterns of VCAM-1 in the endothelium of nondiseased and diseased arteries were similar to those of GATA6, implying that the expression of inflammatory molecule VCAM-1 is also upregulated in the endothelium of human atherosclerotic coronary arteries in vivo.

### 3.4. Low Level of miR-10a in Blood Serum Is Associated with the Development of Human Atherosclerosis

**Figure 4:**
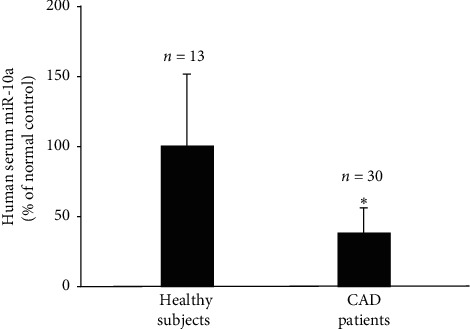
Low level of miR-10a in blood serum is highly related to the progression of human atherosclerotic disease. The levels of miR-10a in blood serum from human subjects without (*n* = 13) or with CAD (*n* = 30) were detected by qPCR. Data show the mean ± SD and are presented as the percentage changes compared to the healthy control groups. ^*∗*^, *P* < 0.05 vs. healthy subjects. CAD, coronary artery disease.

Circulating miR in the blood serum has been identified as a diagnostic component in atherosclerotic disease [16]. Here, we collected blood samples from patients with CAD and healthy subjects to detect the level of miR-10a in their sera. The characteristics of patients with CAD and healthy subjects are shown in Table S1. Results showed that miR-10a level in the blood serum of patients with CAD was significantly lower than that in healthy subjects (Figure 4), indicating that low circulating miR-10a level in the blood serum is associated with the development of human atherosclerosis.

## 4. Discussion

In the present study, we showed that low miR-10a in both cardiovascular endothelium and blood serum is highly related to the development of CAD, i.e., atherosclerosis, and can be used as a potential biomarker for human atherosclerosis, as evident from the findings of this study. First, miR *in situ* hybridization results demonstrated that miR-10a is highly expressed in the endothelial layer of CL arteries and coronary arteries with mini. lesions, but not in the endothelial layer of coronary arteries with advan. lesions. Second, in the endothelial layer of nondiseased human arteries, the lack of transcriptional factor GATA6, which is the direct target of miR-10a, was accompanied by the high level of miR-10a. In contrast, the intense expression of GATA6 in the endothelial layer of diseased coronary arteries was accompanied by the low expression of miR-10a. Third, expression of the inflammatory molecule, VCAM-1, which is the downstream target of miR-10a and GATA6, was downregulated in the endothelial layer of nondiseased arteries, whereas it was upregulated in the endothelial layer of diseased coronary arteries. Fourth, detection of miR-10a in human blood from patients with CAD compared to that from healthy subjects demonstrated that miR-10a level in patient serum is significantly lower than that in serum from healthy subjects. Collectively, our findings provide a new concept that the reduced expression of miR-10a in both cardiovascular endothelium and blood serum may promote human blood vessels toward atherosclerotic lesions in the cardiovascular system.

### 4.1. Low Level of “Atheroprotective miR-10a” in the Endothelium Is Highly Related to the Development of Human CAD, i.e., Atherosclerosis

MiRs have emerged as vital factors affecting cardiovascular pathology [5–8]. Several miRs can be modulated by blood shear to regulate signaling molecules involved in signal transductions that modulate vascular EC dysfunctions, i.e., inflammation, proliferation, and oxidation [5, 8–10]. Notably, Fang et al. used miR microarray to analyze the expressions of various miRs in the atherosclerosis-susceptible region (OS region) relative to those in atherosclerosis-resistant regions (PS region) of the cardiovascular system in an animal swine model and reported that miR-10a was the least expressed miR in the endothelium of OS region, i.e., aortic arch and aorta-renal branches, compared to that in other regions in vivo [11]. Furthermore, they also transfected human umbilical vein endothelial cells (HUVEC) with antagomiR-10a and demonstrated that miR-10a exerts an anti-inflammatory effect by inhibiting NF-*κ*B signaling in ECs [11]. Our previous studies used an in vitro flow system to identify that proatherogenic OS induces a sustained decrease in miR-10a expression, whereas atheroprotective PS induces a sustained increase in miR-10a expression in ECs. By investigating the effect of fluid shear on all the examined shear-sensitive miRs in ECs, our results also showed miR-10a as the OS-modulated miR with the lowest expression [12]. We further used an in vivo diseased ApoE-/- mouse model to demonstrate that endothelial miR-10a is increased in the PS region of the cardiovascular system but inhibited in the OS region. These findings indicated that the precursor miR-10a injection could induce miR-10a expression in the endothelium of blood vessels to inhibit atherosclerotic lesion formation in ApoE-/- mice [12]. In accordance, the present findings demonstrated that miR-10a is undetectable in the endothelium of atherosclerotic lesions in diseased human coronary arteries, in contrast to its high expression in the endothelium of nondiseased arteries. These findings suggest that the low level of “atheroprotective miR-10a” in the endothelium is highly related to the development of human CAD, i.e., atherosclerosis.

### 4.2. Low Level of Endothelial miR-10a Results in an Intense Expression of Transcriptional Factor GATA6 during the Development of Human Atherosclerosis

GATA6 has been identified as the direct target of miR-10a, and its expression can be inhibited by miR-10a binding to 3′-UTR of GATA6 [12]. GATA factors are zinc finger-containing transcriptional factors that bind specific DNA region in the promoter to regulate gene expression [18]. GATA6 is a member of the GATA factor family. It is expressed in a wide range of human tissues, e.g., heart, liver, kidney, pancreas, and spleen, to regulate differential responses in these tissues [19]. In ECs, GATA6 has been defined as the vital transcriptional factor for inflammatory signaling [17]. Our previous study has shown that the expression of GATA6 can be modulated by shear-regulated miR-10a in the ECs. Atheroprotective PS induces miR-10a to inhibit the expression of its direct target GATA6, whereas atherogenic OS represses miR-10a to turn on the expression of GATA6 in the ECs in vitro [12]. In vivo results using ApoE-/- mice showed that GATA6 is overexpressed in the endothelium of atherosclerosis-susceptible region (OS region) of the cardiovascular system, but not in the endothelium of atherosclerosis-resistant region (PS region). Injection of ApoE-/- mice with miR-10a precursor inhibited the GATA6 expression in the endothelium of atherosclerosis-susceptible region [12]. The present study further extended these findings in the development of human atherosclerotic disease. The present study demonstrated that the inhibition of GATA6 is accompanied by the high expression of miR-10a in the endothelium of human nondiseased arteries. In contrast, the intense expression of GATA6 is accompanied by the reduced expression of miR-10a in the endothelium of human diseased coronary arteries. Taken together, our findings suggest that a low level of endothelial miR-10a results in an intense expression of GATA6 during the development of human atherosclerotic disease.

### 4.3. Low miR-10a Level Induces Transcriptional Factor GATA6 to Upregulate Inflammatory VCAM-1 during the Development of Human Atherosclerosis

Cell adhesion molecules are involved in cell-cell interaction [20]. In atherogenesis, ECs induce the expression of cell adhesion molecule, i.e., VCAM-1, to facilitate the adherence of monocytes to the endothelium as well as their infiltration into the vessel wall at the initiation of atherosclerosis [21]. Thus, VCAM-1 is an important cell adhesion molecule for the initiation of atherosclerosis [21, 22]. VCAM-1 has been identified to be regulated transcriptionally by the transcriptional factor GATA6 in ECs in response to TNF*α*, and its promoter region contains the GATA6-binding site [17, 23]. Our previous results have shown that VCAM-1 can be regulated transcriptionally by GATA6 in ECs in response to blood fluid flow. Moreover, PS and OS can alter EC miR-10a levels to modulate the expression of GATA6 and subsequently regulate the expression of its downstream molecule VCAM-1 in vitro [12]. Our in vivo studies on the diseased ApoE-/- mouse model showed that VCAM-1 is upregulated in the endothelium of the OS region in the cardiovascular system but not in that of the PS region. Furthermore, it has also been shown that endothelial miR-10a induction by injection of miR-10a precursor can inhibit GATA6/VCAM-1 signaling [12] and inflammatory cell infiltration in the atherosclerotic vessel wall in ApoE-/- mice [13]. In the present study, we found VCAM-1 to be upregulated in the endothelium of human diseased coronary arteries but downregulated in that of human nondiseased arteries. The expression patterns of VCAM-1 in human nondiseased arteries vs. human diseased coronary arteries were similar to those of the transcriptional factor GATA6. Taken together, these results imply that a low miR-10a level induces an intense expression of the transcriptional factor GATA6 to upregulate the expression of the inflammatory molecule VCAM-1 during the development of human atherosclerotic disease.

### 4.4. Low Levels of Circulating miR-10a in Human Subjects Are Related to Atherogenesis

Circulating miR has emerged as a new diagnostic component in cardiovascular diseases [16, 24–26]. Our previous study using the ApoE-/- mouse model has shown that a repressed level of serum circulating miR-10a is associated with the development of atherosclerosis [13]. Our present study demonstrated that the level of circulating miR-10a in the serum from patients with CAD is significantly lower than that in normal human subjects. These results suggest that low levels of circulating miR-10a in human subjects are related to the development of human CAD, such as atherosclerotic disease.

## 5. Conclusions

Our findings identify the relationship between miR-10a signaling and CAD, i.e., atherosclerosis in humans. Low miR-10a and high GATA6/VCAM-1 levels in the cardiovascular endothelium are highly related to the pathobiology of human atherosclerotic lesions. The reduced expression of endothelial miR-10a seems to enhance the expression of downstream GATA6/VCAM-1 to turn on inflammatory signaling and promote human blood vessels toward atherosclerotic lesions. The expression pattern of low miR-10a and high GATA6/VCAM-1 in cardiovascular endothelium can be helpful as a diagnostic biomarker for atherosclerotic lesions. In addition, we also demonstrated that patients with CAD have a significantly lower concentration of miR-10a in their serum compared with healthy subjects. The low serum level of miR-10a can potentially be used to develop new diagnostic strategies for human atherosclerotic diseases. Our study provides novel information regarding miR-10a in relation to human CAD.

## Data Availability

The data used to support the findings of this study are included within the article.

## Supplementary Materials

Supplementary Materials Table S1: the characteristics of healthy subjects and CAD patients.
