# Supplementary material for: Low Levels of MicroRNA-10a in Cardiovascular Endothelium and Blood Serum Are Related to Human Atherosclerotic Disease
Source: Cardiol Res Pract. 2021 Jul 15;2021:1452917. doi: 10.1155/2021/1452917 (PMC8298183; doi:10.1155/2021/1452917)
Supplement: Supplementary Materials — Table S1: the characteristics of healthy subjects and CAD patients. [file 1452917.f1.doc]

**Table S1. Characteristics of healthy subjects and CAD patients.**

|  | **Healthy subjects (n=13)** | | |  | | **CAD Patients (n=30)** | | | | |
| --- | --- | --- | --- | --- | --- | --- | --- | --- | --- | --- |
| **Stable CAD for at least 3 months** |  | 0 |  | |  | |  | 30 |  |  |
| **Age (years)** |  | 37.46±8.5 |  | |  | |  | 66.43±11.12 |  |  |
| **Gender Male** |  | 4 |  | |  | |  | 20 |  |  |
